# Supplementary material for: Understanding the Polyamine and mTOR Pathway Interaction in Breast Cancer Cell Growth
Source: Med Sci (Basel). 2022 Sep 10;10(3):51. doi: 10.3390/medsci10030051 (PMC9504347; doi:10.3390/medsci10030051)
Supplement: Supplementary file 1 [file medsci-10-00051-s001.zip › supplementary materials.pdf]

medical sciences

MDPI

Communication

Understanding the Polyamine and mTOR Pathway Interaction in Breast Cancer Cell Growth

Oluwaseun Akinyele [1,2] and Heather M. Wallace [1,*]

[1] Institute of Medical Sciences, School of Medicine, Medical Sciences and Nutrition, University of Aberdeen, Aberdeen, AB25 2ZD, Scotland, UK
[2] Div. of Genetics and Genomic Medicine, Dept. of Pediatrics, University of Pittsburgh School of Medicine, Pittsburgh, PA, 15260, USA
* Correspondence: h.m.wallace@abdn.ac.uk

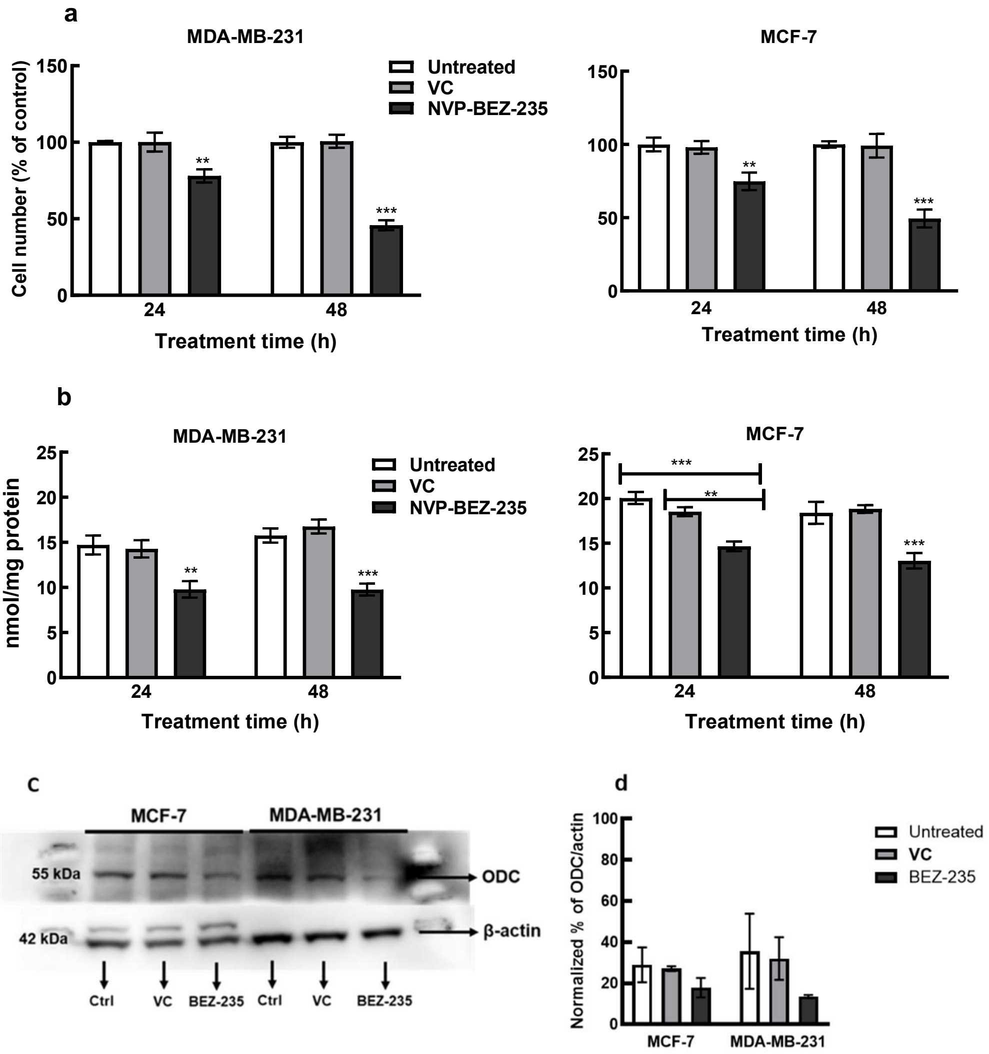

Figure S1. Effect of NVP-BEZ-235 on the growth, polyamine content and ODC protein expression in the breast cancer cells. Cells were seeded for 24 h. Thereafter, MDA-MB-231 and MCF-7 cells were treated with 10 μM and 1 μM NVP-BEZ-235 respectively and DMF as vehicle control, VC. (a) Cell number was determined by trypan-blue exclusion assay. (b) Total polyamine content was determined by LC-MS. (c) ODC expression was determined by Western blot. (d) Densitometry of ODC and β-actin from western blot images. Results shown are mean ± S.E.M for three independent experiments and two replicates per treatments or mean± range for two independent experiments. Results were analysed using ANOVA and Dunnett post-test where $^{**}P < 0.01$, $^{***}P < 0.001$ and were considered statistically significant. **Note**: BEZ-235 in c and d represents NVP-BEZ-235 in a and b.

medical sciences

MDPI

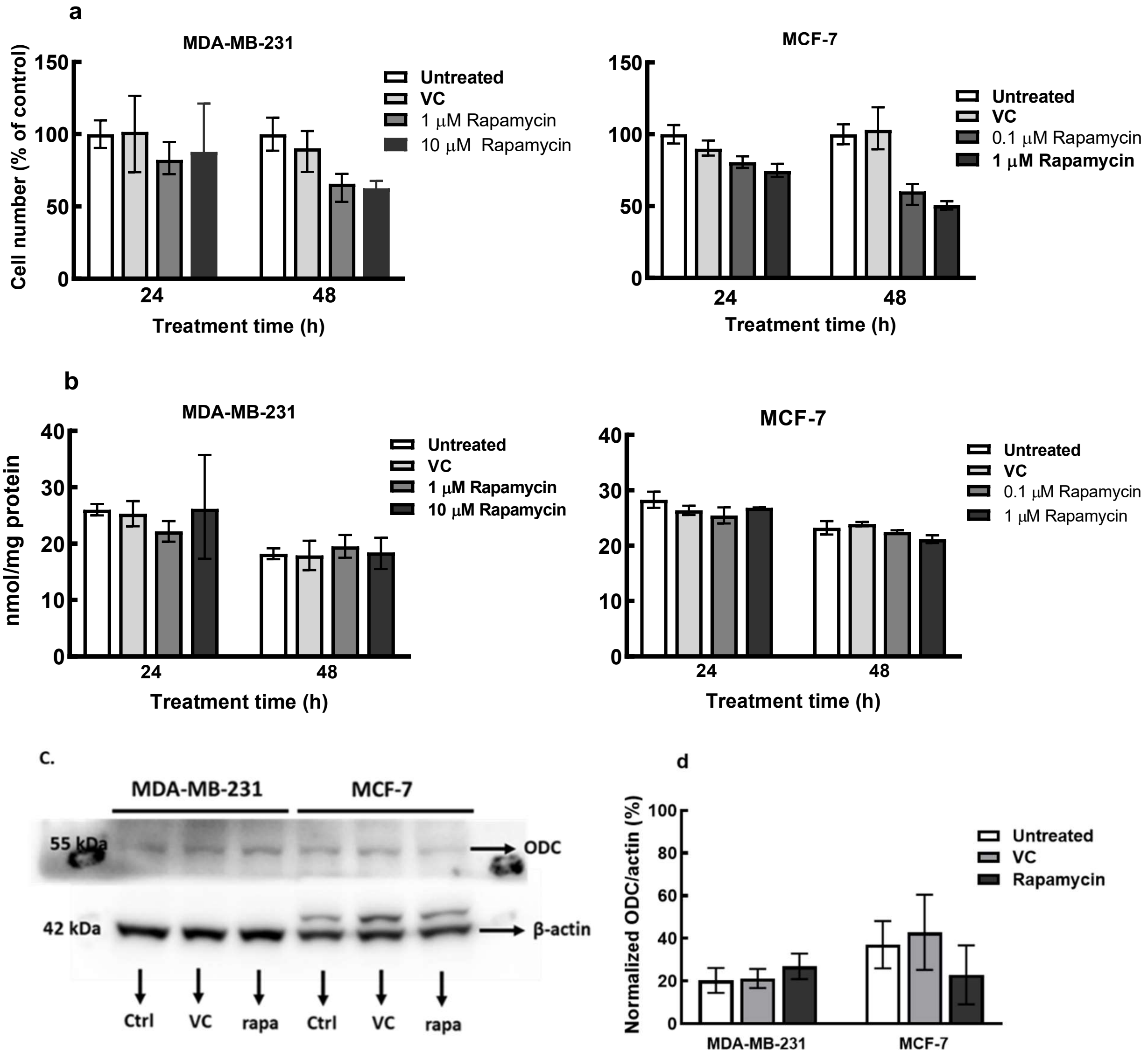

Figure S2. Effect of rapamycin on the growth, polyamine content and ODC protein expression in the breast cancer cells. MDA-MB-231 and MCF-7 cells were seeded for 24 h. Thereafter cells were treated with respective concentrations of rapamycin, DMSO was used as vehicle control, VC. (a) Cell number was determined by trypan-blue exclusion assay. (b) Total polyamine content was determined by LC-MS. (c) ODC expression was determined after 48 h exposure of MDA-MB-231 and MCF-7 to 10 μM and 1 μM Rapamycin respectively. (d) Densitometry of ODC and β-actin from western blot image. Results shown are mean ± range for two independent experiments. **Note**: rapa in c represents rapamycin in a, b and d.
