# Supplementary figures and images for: Understanding the Polyamine and mTOR Pathway Interaction in Breast Cancer Cell Growth
Source: Med Sci (Basel). 2022 Sep 10;10(3):51. doi: 10.3390/medsci10030051 (PMC9504347; doi:10.3390/medsci10030051)

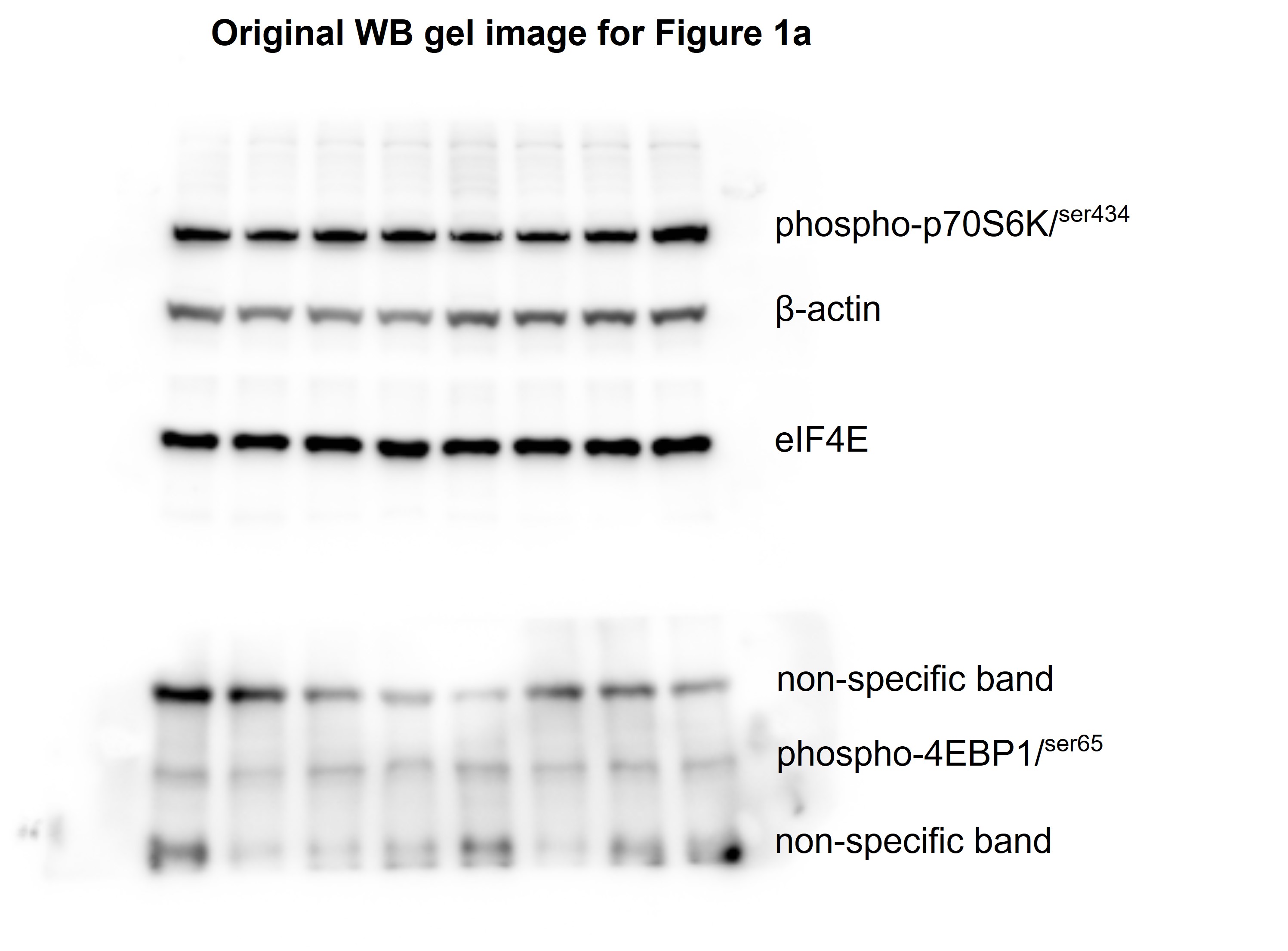

Supplement: Supplementary file 1 [file medsci-10-00051-s001.zip › Original WB images/Original WB for Figure 1a.jpg]

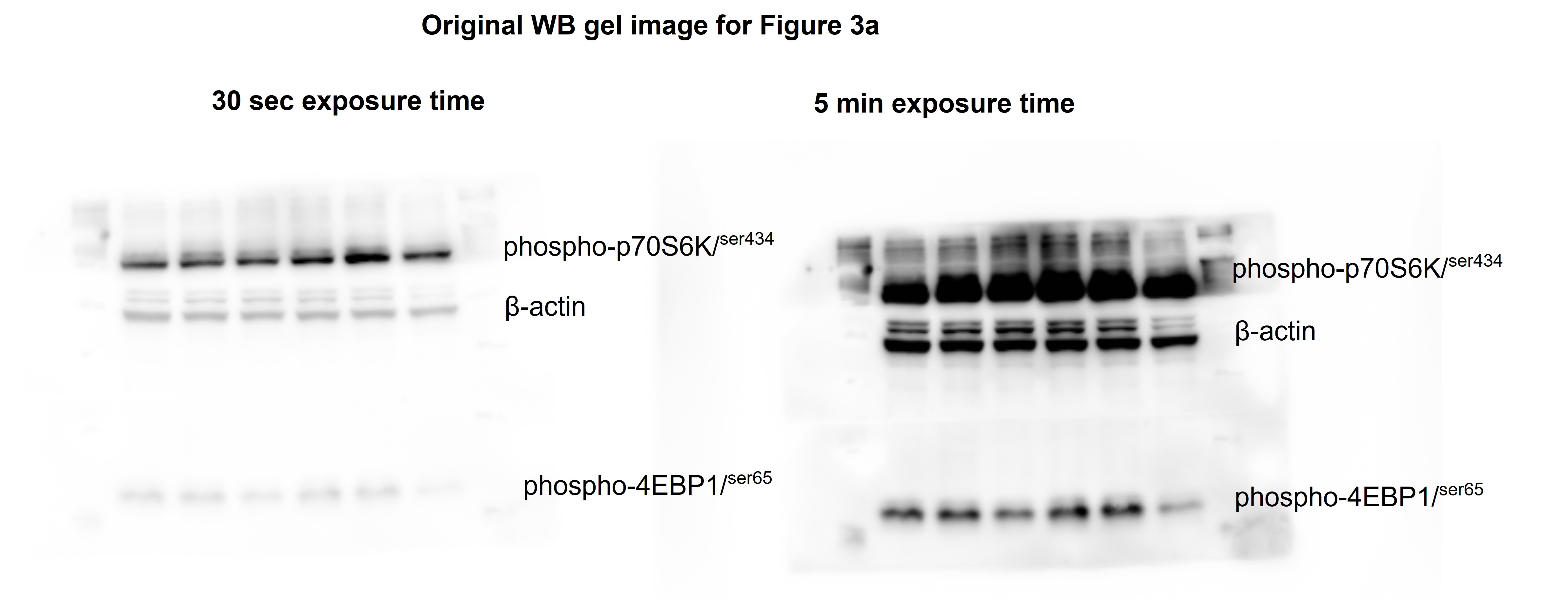

Supplement: Supplementary file 1 [file medsci-10-00051-s001.zip › Original WB images/Original WB for Figure 3a.jpg]

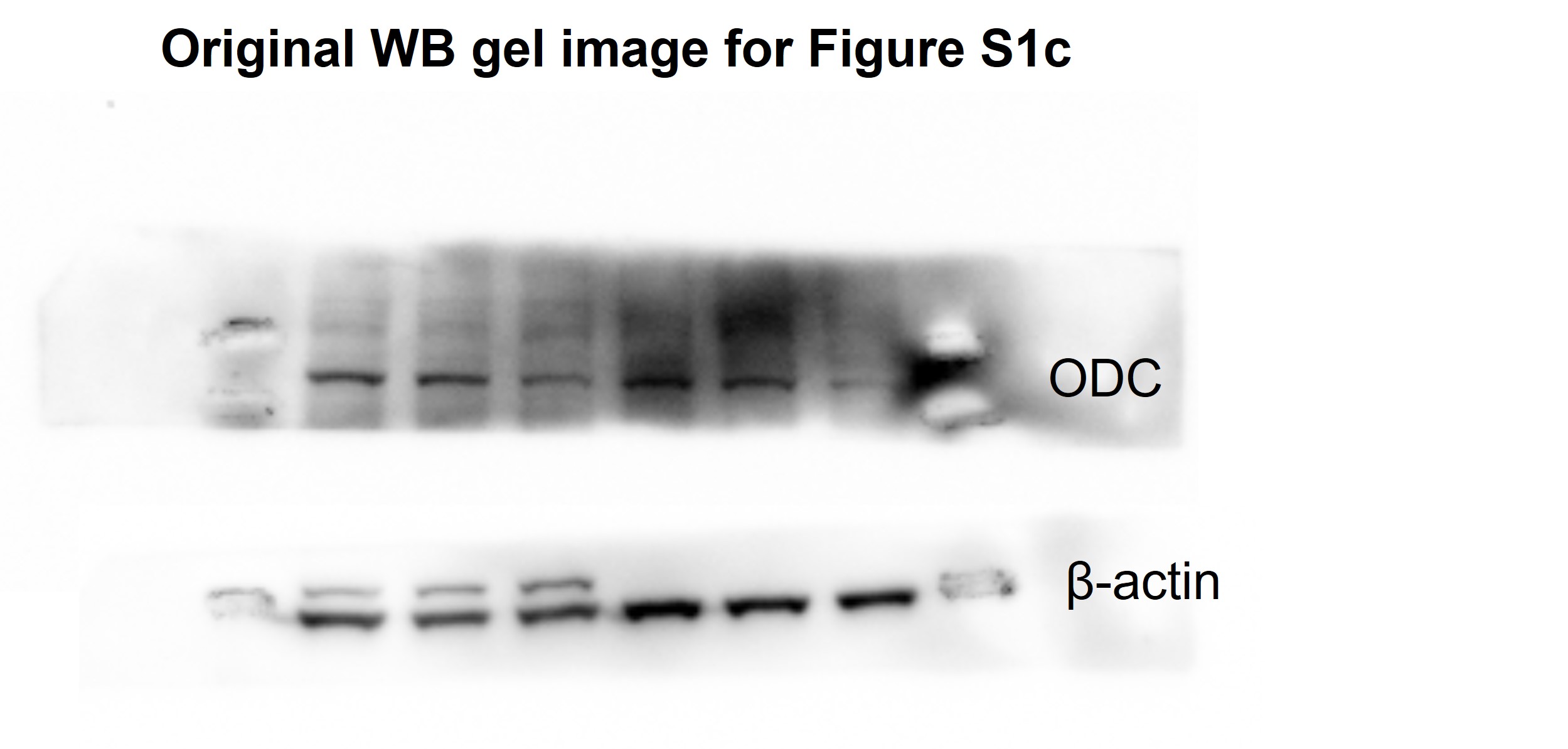

Supplement: Supplementary file 1 [file medsci-10-00051-s001.zip › Original WB images/Original WB for Figure S1c.jpg]

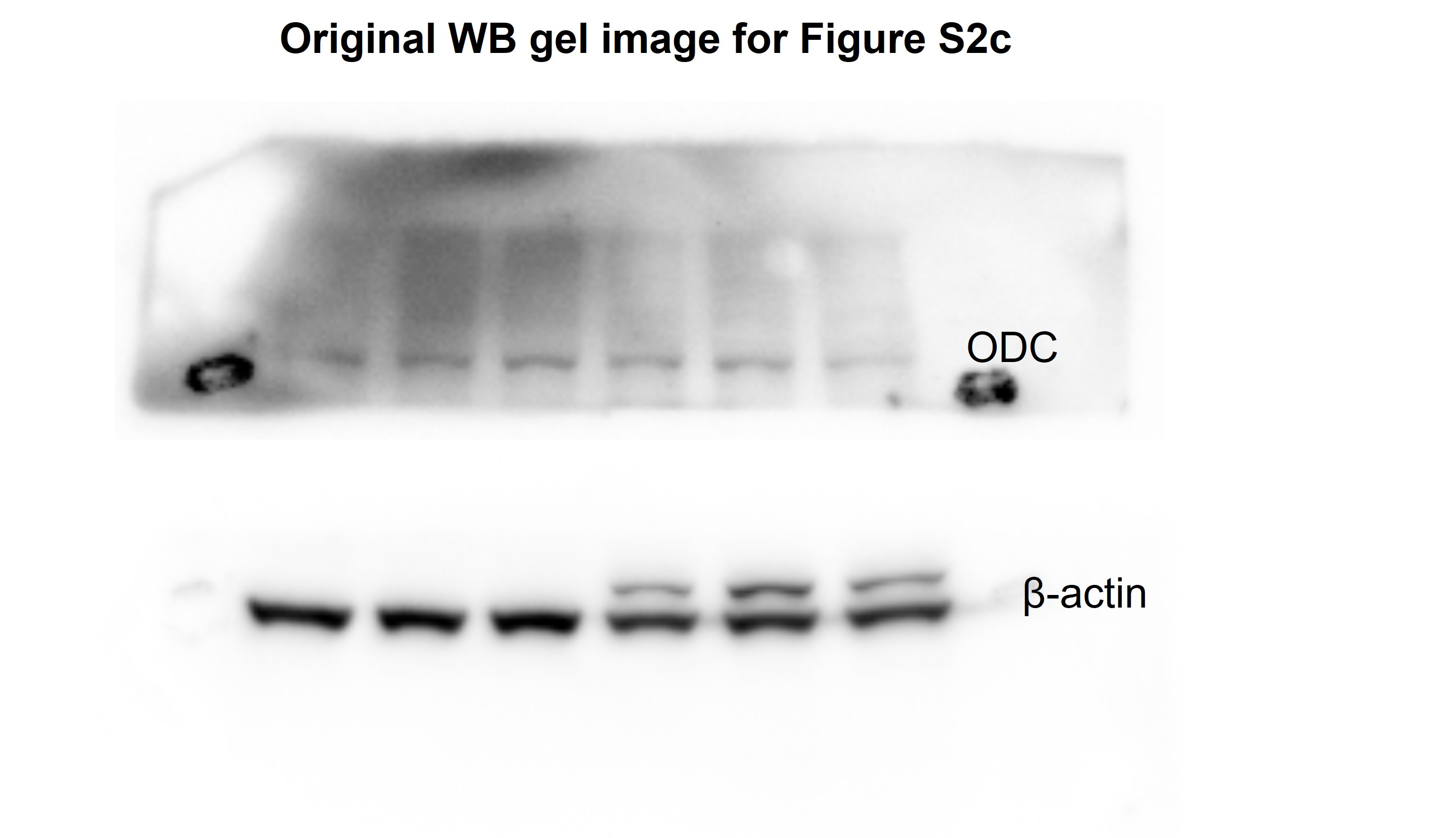

Supplement: Supplementary file 1 [file medsci-10-00051-s001.zip › Original WB images/Original WB for Figure S2c.jpg]
